# Structure and Function of p97 and Pex1/6 Type II AAA+ Complexes

**DOI:** 10.3389/fmolb.2017.00033

**Published:** 2017-05-29

**Authors:** Paul Saffert, Cordula Enenkel, Petra Wendler

**Affiliations:** ^1^Department of Biochemistry, Institute of Biochemistry and Biology, University of PotsdamPotsdam, Germany; ^2^Department of Biochemistry, University of TorontoToronto, ON, Canada

**Keywords:** type II AAA+ ATPases, Pex1, Pex6, p97, cryo electron microscopy

## Abstract

Protein complexes of the Type II AAA+ (ATPases associated with diverse cellular activities) family are typically hexamers of 80–150 kDa protomers that harbor two AAA+ ATPase domains. They form double ring assemblies flanked by associated domains, which can be N-terminal, intercalated or C-terminal to the ATPase domains. Most prominent members of this family include NSF (N-ethyl-maleimide sensitive factor), p97/VCP (valosin-containing protein), the Pex1/Pex6 complex and Hsp104 in eukaryotes and ClpB in bacteria. Tremendous efforts have been undertaken to understand the conformational dynamics of protein remodeling type II AAA+ complexes. A uniform mode of action has not been derived from these works. This review focuses on p97/VCP and the Pex1/6 complex, which both structurally remodel ubiquitinated substrate proteins. P97/VCP plays a role in many processes, including ER- associated protein degradation, and the Pex1/Pex6 complex dislocates and recycles the transport receptor Pex5 from the peroxisomal membrane during peroxisomal protein import. We give an introduction into existing knowledge about the biochemical and cellular activities of the complexes before discussing structural information. We particularly emphasize recent electron microscopy structures of the two AAA+ complexes and summarize their structural differences.

## Introduction

The conversion of chemical energy in the form of nucleotide triphosphates into mechanical energy is a process utilized by all living cells and associated with a large variety of cellular functions. Proteins of the AAA+ superfamily are often essential parts of such molecular machines. They catalyze the hydrolysis of ATP to ADP resulting in mechanical work on a substrate molecule. To date, at least 80.000 AAA+ domains (Pfam ID: PF00004; Finn et al., 2016) have been identified throughout more than 5400 species covering all kingdoms of life. The protein data bank stores structures of 722 proteins with AAA+ domains. All AAA+ have a structurally conserved nucleotide-binding domain (NBD) in common, usually comprising 200–250 amino acids (AA), which is essentially responsible for ATP binding and subsequent hydrolysis (Wendler et al., 2012). All AAA+ NBDs share a conserved Rossmann fold domain with a 51432 topology of the central β-sheet and a C-terminal alpha helical domain. They contain multiple conserved features including the Walker A motif, Walker B motif (Walker et al., 1982) and the second region of homology (SRH) (Swaffield et al., 1992). The SRH, differentiating the classic AAA proteins from other Walker A/B ATP binding proteins, typically contains Arg-residues, which work *in trans* in the active hexamer to facilitate ATP-hydrolysis by interacting with the γ-phosphate of ATP bound to the neighboring subunit (Karata et al., 1999; Neuwald et al., 1999). The remaining sequence, N-terminal and C-terminal to the NBD, often show very little sequence homology among the AAA+ protein family. The AAA+ family can be further classified by the presence of one NBD (type I) or two consecutive NBDs (type II; NBD1 and NBD2). This review will focus on comparing recent EM structures of two prominent candidates of the type II AAA+ class, p97 (Cdc48, yeast homolog) and the Pex1/Pex6 complex, both belonging to the classic clade of AAA+ proteins (Iyer et al., 2004). The two complexes are the only known members of the class to be involved in remodeling of ubiquitinated substrates, although none of the ATPases harbors a high affinity ubiquitin interaction domain. Intriguingly, the mode of action of the AAA+ complexes is also highly debated and possibly differs between the two functional and structural homologs. The scope of this review is not to give an exhaustive summary of the structural data or the cellular role of the complexes. There are many excellent reviews on the cellular role, on co-factor binding, and on the structure of p97 (Meyer et al., 2012; Buchberger, 2013; Olzmann et al., 2013; Dantuma et al., 2014; Tang and Xia, 2016; Xia et al., 2016) or Pex1/Pex6 (Fujiki et al., 2012; Waterham and Ebberink, 2012; Grimm et al., 2016). We would like to refer the interested reader to these publications for an in-depth coverage of these topics.

## Cellular functions of the p97 protein complex

P97 is an essential protein with a broad cellular distribution. It makes up 1% of the total cellular protein pool and is one of the most conserved proteins in eukaryotes emphasizing its importance in cell homeostasis (Wang et al., 2004). Moir et al. first described the yeast variant of p97 in 1982 and it was preliminarily associated with cell cycle arrest, thus called Cdc48 (Cell Division Cycle) (Moir et al., 1982). Since then, p97 has been intensively investigated and implicated in a myriad of functions. The best documented role of p97 has been established in the Ubiquitin-Proteasome-System (UPS) for mobilizing target proteins for degradation by the 26S proteasome (Ghislain et al., 1996; Hitchcock et al., 2001; Rape et al., 2001; Ye, 2006; Jentsch and Rumpf, 2007; Stolz et al., 2011), in particular during endoplasmic-reticulum-associated degradation (ERAD) (Ye et al., 2001; Jarosch et al., 2002; Rabinovich et al., 2002; Meusser et al., 2005). Over the last decade p97's critical role has been furthermore associated with cell-cycle regulation (Meyer and Popp, 2008; Meyer et al., 2012) and DNA repair (Meerang et al., 2011; Ramadan and Meerang, 2011). Due to the above mentioned diverse functions, p97 has been termed “the Swiss army knife of cell biology” (Baek et al., 2013) and “a molecular gearbox” in the ubiquitin pathway (Jentsch and Rumpf, 2007). These different tasks are enabled and regulated by many adaptors/clients, which recruit and connect p97 to different cell organelles (Dreveny et al., 2004). Furthermore, the capability of p97 associating with ubiquitinated substrates as well as ubiquitin-binding adaptors/clients adds to its versatility (Rape et al., 2001; Richly et al., 2005). The N-terminal domain predominantly mediates adaptor/client binding, however numerous binding partners for the C-terminus have been identified (Buchberger et al., 2015).

So far, at least 40 proteins have been found to interact with p97 in mammalian systems. Intriguingly, most of these factors share common binding motifs and conserved binding modules (Buchberger et al., 2015). Binding and recruitment of adaptors/clients to the N-terminus is facilitated via the ubiquitin related UBX-domain (ubiquitin regulatory X) or UBXL-domain (UBX-like) and the three linear motifs named VIM (VCP interacting motif), VBM (VCP binding motif) or SHP box (Yeung et al., 2008). The number and variety of different interaction motifs suggest a high temporal and spatial regulation of interacting partners. To date, 13 human proteins have been identified possessing an UBX-domain, all of which have been implicated in p97 binding (Yeung et al., 2008; Buchberger et al., 2015). All these proteins compete for the same binding domain in the N-terminus of p97. It has been shown that the nucleotide binding state of p97 can be a discriminating factor for binding different adaptors/clients. The Ufd1/Npl4 heteromeric complex possesses a SHP box (Ufd1) and an UBXL-domain (Npl4), respectively, and recruits substrates of proteasomal degradation or processing pathways to p97. In contrast, p47 possesses an SHP box as well as an UBX-domain and recruits non-proteasomal substrates to p97. Binding of ATP rather than ADP in the first nucleotide-binding-domain increases the association of the Ufd1/Npl4/p97 complex, allowing for competition with p47, thereby regulating the engagement of p97 in either directed proteasomal proteolysis or non-proteasomal proteolysis pathways (Chia et al., 2012). In addition to the interaction with the N-terminal domain, multiple reports highlight the finding that the C-terminal tail of NBD2 is also capable of binding to specific substrates. This interaction has so far been shown for proteins containing a PUB (PNGase/UBA or UBX containing proteins) or PUL (PLAP, Ufd3p, and Lub1p) domain (Allen et al., 2006; Qiu et al., 2010; Chia et al., 2012). PUB-domain proteins can bind to the C-terminal PIM-motif (PUB-interacting motif) of p97 whereas the interaction with the PUL-domain is more controversial (Zhao et al., 2009; Qiu et al., 2010). Biochemical and mutational analysis determined the binding site of Cdc48 on the yeast Plaa homolog Doa1 (Zhao et al., 2009), but a crystal-structure of the human Plaa with the 10 AA C-terminal peptide of p97 suggest a different binding pocket (Qiu et al., 2010). Intriguingly, the protein UBXD1 can interact with both termini by two independent binding sites, thus being a very unique co-factor of p97 (Kern et al., 2009). Binding studies are complicated by the high oligomeric organization of p97 with a total of 6 N-termini in the complex, thereby allowing theoretically 6 individual binding partners. The matter is further complicated by the fact that many co-factors possess more than one binding motif and that some of the above described binding sites are overlapping, i.e., VIM/VBM and UBX/UBXL. To summarize, in order to understand the entire network of regulatory interactions between different adaptors/clients and p97 further investigation is needed.

## ATPase activity of the p97 complex

A unique feature of p97 distinguishing it from the other type 2 AAA+ proteins is the high level of conservation of the two individual NBD's. Although the two domains share over 40% identity at sequence level, multiple studies have shown that the two domains of p97 contribute differently to the bulk hydrolysis activity. Nucleotide binding by NBD1 accelerates but is not required for p97 oligomerization (Wang et al., 2003). Once the hexamer is formed, full length p97 appears unable to exchange nucleotide in NBD1 at physiological temperatures (Davies et al., 2005). In contrast, NBD2 is the major site of ATP hydrolysis in the p97 complex (Song et al., 2003). Yet, NBD1 possesses an intrinsic hydrolysis activity and has been linked to the heat-shock induced ATP activity observed with p97/Cdc48 (Song et al., 2003). Binding studies using ITC performed with purified murine p97 have revealed that NBD1 of p97 has a 90-fold higher binding affinity toward ADP compared to NBD2 (Kd_NBD1_ = 1 μM; Kd_NBD2_ = 90 μM; Table 1). Interestingly, the binding affinities for the two domains toward the ATP-analog ATPγS are with K_d_ = 2 μM and K_d_ = 3 μM for NBD1 and NBD2, respectively, very similar (Briggs et al., 2008). ATPγS has been a proven valuable ATP analog for studying binding kinetics and simultaneously excluding residual hydrolysis activity. In contrast to other analogs, it shows similar binding kinetics as ATP. Intriguingly, even with saturating amounts of nucleotide only 9–10 of the feasible NBDs are occupied (Briggs et al., 2008). ATPγS binding to the NBD2 ring of p97 shows a Hill-coefficient of 3–4, implying that 3–4 protomers of the complex positively cooperate upon ATP binding. It has to be highlighted that different groups have determined diverging K_m_-values for ATP hydrolysis ranging from 3 to 620 μM (Meyer et al., 1998; DeLaBarre et al., 2006; Briggs et al., 2008; Nishikori et al., 2011; Niwa et al., 2012). Most of these studies have investigated steady-state kinetics. The variations can be partly accounted for by the different homologs and conditions utilized. Several explanations for coordinated ATPase activity in the AAA+ ring have been proposed. For the NBD2 ring of p97, a concerted ATPase hydrolysis seems unlikely since most reports mention unequal nucleotide occupancy in the ring. The concerted approach states that all 6 NBD2 bind, hydrolyze and release nucleotide with identical parameters. It is more plausible to assume that ATP hydrolysis in the NBD2 ring has a “binding change” mechanism, i.e., binding of ATP to one NBD2 positively influences binding of ATP, ADP release or hydrolysis in the adjacent NBD2. Thus, the ATP hydrolysis would proceed in a rotary manner. The positive cooperativity of ATP binding observed in several p97 species favors this mechanism over the possible random ATP hydrolysis (DeLaBarre et al., 2006; Briggs et al., 2008; Nishikori et al., 2011).

**Table 1:** **Summary of the conserved motifs as well as the effects of reported mutations in these motifs of the type II AAA+ proteins ***Homo sapiens (hs)*** p97, ***Saccharomyces cerevisiae*** (sc) Cdc48, ***Thermoplasma acidophilum (ta)*** p97/VAT and the two peroxins Pex1/Pex6 from ***S. cerevisiae*****.

		***hs*****p97**			***sc*****Cdc48**			***ta*****p97/VAT**			***sc*****Pex1**			***sc*****Pex6**		
**NBD 1**	**Walker A**	**GPPGTGKT**			**GPPGTGKT**			**GPPGTGKT**			**GKQGIGKT**			**TTNNVGKA**		
		GPPGTGTT: (Song et al., 2003; Wang et al., 2004)	ATPase	++	GPPGTGAT (Esaki and Ogura, 2010)	Function	−	GPPGTGAT (Gerega et al., 2005)	ATPase	+	GKQGIGTT (Birschmann et al., 2005; Gardner et al., 2015)	Function	+++	TTNNVGT/AA (Birschmann et al., 2003)	Function	+++
			Function	++	GPPGTGTT (Esaki and Ogura, 2010)	Function	+		Function	−						
	**Walker B**	**IIFIDE**			**IIFIDE**			**IIFIDE**			**LIVLDN**			**VIFLAH**		
		IIFIDQ (Song et al., 2003)	ATPase	++	IIFIDQ (Esaki and Ogura, 2010)	Function	−	IIIFDA (Gerega et al., 2005)	ATPase	+	LIVLQN (Platta et al., 2005)	Function	+++	n.d.		
									Function	+						
	**Arg-Finger**	**RFGR**			**RFGR**			**RFGR**			**n.d**.			**RSHMR**		
		AFGR (Wang et al., 2005)	ATPase	++	AFGR (Esaki and Ogura, 2010)	Function	++	n.d.			n.d.			KSHMR (Ciniawsky et al., 2015)	Function	++
			Function	−												
		RFGA (Wang et al., 2005)	ATPase	++										RSHMK (Ciniawsky et al., 2015)	Function	+++
			Function	−												
**NBD2**	**Walker A**	**GPPGCGKT**			**GPPGTGKT**			**GPPGVGKT**			**GYPGCGKT**			**GPPGTGKT**		
		GPPGTGTT: (Song et al., 2003; Wang et al., 2004)	ATPase	+	GPPGTGA/TT (Esaki and Ogura, 2010)	Function	−	GPPGVGAT (Gerega et al., 2005)	ATPase	+	GYPGCGET (Birschmann et al., 2005; Platta et al., 2005)	Function	−	GPPGTGAT (Birschmann et al., 2005; Platta et al., 2005)	Function	−
			Function	+					Function	+						
	**Walker B**	**VLFFDE**			**VVFLDE**			**IVFLDE**			**ILFFDE**			**VIFFDE**		
		VLFFDQ (Song et al., 2003)	Function	−	VVFLDQ (Esaki and Ogura, 2010)	Function	−	IVFLDA (Gerega et al., 2005)	ATPase	++	ILFFDQ (Ciniawsky et al., 2015)	ATPase	++	VIFFDQ (Ciniawsky et al., 2015)	ATPase	−
									Function	++						
	**Arg-Finger**	**RPGR**			**RPGR**			**RAGR**			**RPGR**			**RPGR**		
		A/KPGR (Wang et al., 2005)	Function	−	APGR (Esaki and Ogura, 2010)	Function	−	n.d.			RPGK (Ciniawsky et al., 2015)	ATPase	–	RPGK (Ciniawsky et al., 2015)	ATPase	–
		RPGA/K (Wang et al., 2005)	Function	−								Function	−		Function	–
**ATP affinity**		NBD1: 2 μM NBD2: 3 μM (Briggs et al., 2008)			n.d.			n.d.			n.d.			n.d.		
**ATP affinity**		NBD1: 2 μM NBD2: 3 μM (Briggs et al., 2008)			550 μM (Fröhlich et al., 1995)			220-350 μM (Gerega et al., 2005)			n.d.			170 μM–690 μM (Saffian et al., 2012; Gardner et al., 2015)		
**Binding partners**		40 identified so far						Proteasome			n.d.			Pex15		

*The effects are divided into ATPase activity (ATPase) and functionality (Function). Functionality either refers to specific functions measured in vitro and in vivo or in the case of the S. cerevisiae proteins: growth phenotype. The amino acid sequences of the conserved motifs are given in one-letter code. Mutated amino acids are underlined. +++, wild type (wt) activity; ++, 50–100% of wt activity: +, 50%–0% of wt activity; −, no measureable activity/function; n.d., not determined*.

It appears that the type of nucleotide bound to NBD1 has a vital role in the overall ATPase activity. As mentioned above, the NBD1 is mainly responsible for efficient hexamerization in a nucleotide dependent manner. However, a Walker B mutation in NBD1 (NBD1^E305Q^), trapping the domain in a permanent ATP bound state, results in 2-fold decrease in the ATPase activity while having the same apparent Km value for ATP (DeLaBarre et al., 2006; Nishikori et al., 2011). Interestingly, this mutation in the yeast homolog Cdc48 causes a lethal phenotype (Table 1). The ATP-trapped Walker B mutant in *Caenorhabditis elegans* Cdc48 affects the overall ATPase activity and negatively influences the cooperative ATPase activity in NBD2 (Nishikori et al., 2011). It has to be mentioned that other mutations inhibiting Cdc48 NBD1 ATPase activity, e.g., Arg-fingers, do not cause a severe phenotype and do not disturb the ATPase activity in the NBD2 ring (Esaki and Ogura, 2010; Nishikori et al., 2011). The effect of NBD1 mutations is controversial and still highly debated, as there are contradictive findings possibly due to the differences between the investigated homologs. Although challenging, measurements of ATPase activity in different mutants have provided valuable evidence for exchange of information between NBDs in one protomer and between neighboring domains in the NBD rings (Briggs et al., 2008; Nishikori et al., 2011; Chou et al., 2014). However, the *in vivo* function is most likely regulated by substrate interaction in combination with specific adaptors/clients and there are only few studies that investigate complex activity in the presence of both. DeLaBarre and co-workers have demonstrated that adding a specific substrate of p97, i.e., the cytoplasmic fragment of Synaptotagmin (Syt1), can substantially increase the basal ATPase activity by approximately 4-fold (DeLaBarre et al., 2006). It will thus be interesting and necessary to correlate structural studies with biochemical results, both in the presence and absence of substrate.

## Cellular function of the Pex1/Pex6 protein complex

Research on peroxisomes (formerly called microbodies) started in the mid 1950's, but the term “peroxisome” was only introduced in 1966 when microbodies were discovered to be important sites of hydrogen peroxide metabolism (De Duve and Baudhuin, 1966). It was not until 1978 when Paul Lazarow described the β-oxidization of fatty acids occurring in peroxisomes (Lazarow, 1978). Further research has established that peroxisomes are also responsible for bile acid biosynthesis, plasmalogens biosynthesis, and compartmentalized catalase glutathione S-transferase activity (reviewed in Morel et al., 2004; Schrader and Fahimi, 2004; Wanders and Waterham, 2006). So far, over 70 distinct proteins have been found in or associated with mammalian peroxisomes. In humans, at least 14 of those proteins are involved in peroxisome biogenesis (Braverman et al., 2016). Mutations in any of these so-called peroxins and in particular in Pex1 and Pex6 have been reported to cause peroxisome biogenesis disorders (PDB), a spectrum of fatal rare diseases (Geisbrecht et al., 1998; Waterham and Ebberink, 2012).

**Figure 1:**
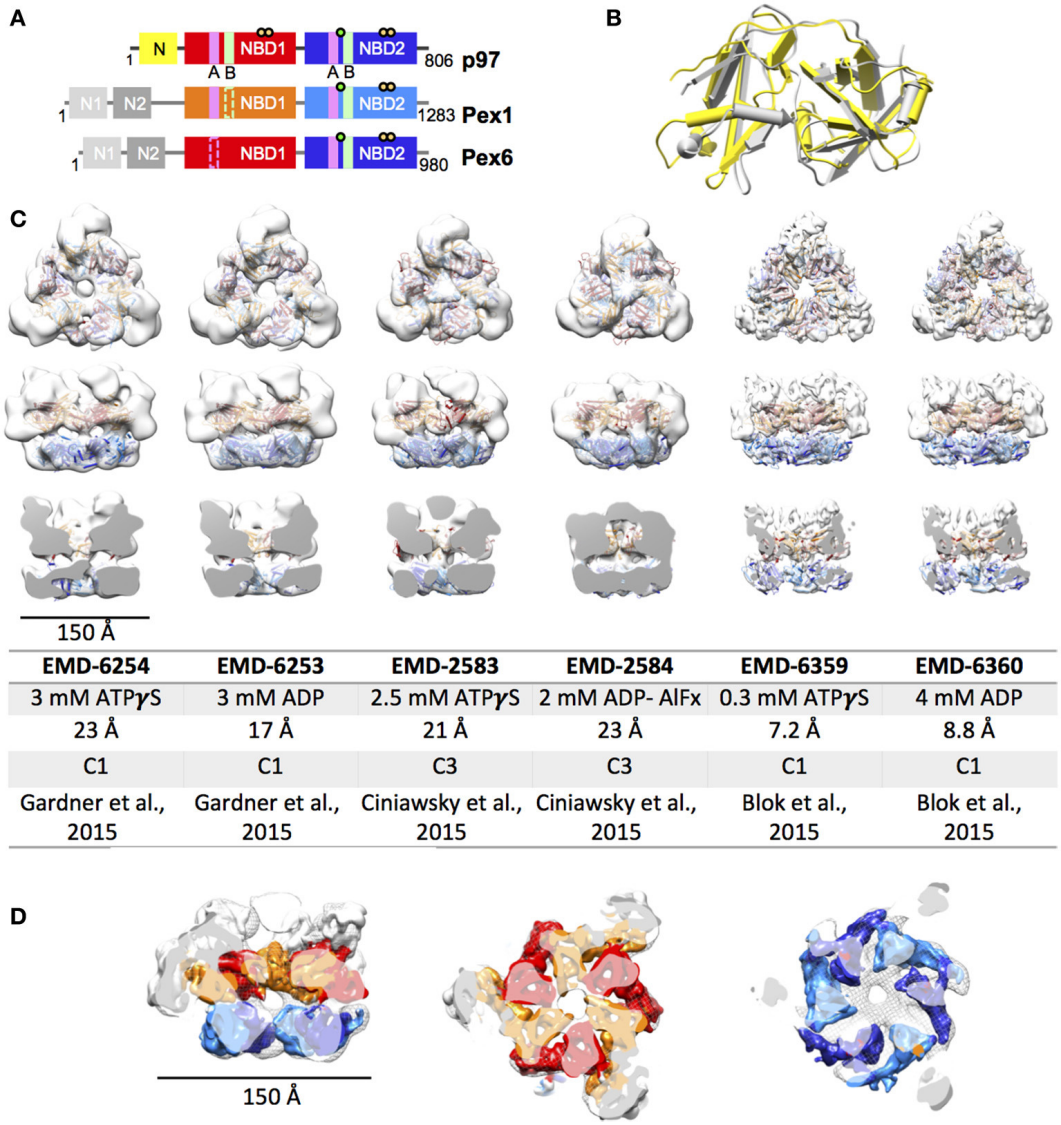
**EM structures of Pex1/6. (A)** Schematic representation of human p97, Pex1 and Pex6. Individual domains and motifs are depicted: N-terminus (N, N1, N2), nucleotide binding domain 1 (NBD1), nucleotide binding domain 2 (NBD2), Walker A (A, magenta bars), Walker B (B, light green bars), Arginine fingers (yellow circle) and substrate binding loops (green circle). Derivation from the canonical motifs is indicted by dotted lines. **(B)** Overlay of the crystal structures of mouse Pex1 (gray, PDB: 1wlf) and mouse p97 (yellow, PDB: 1e32) N-termini. **(C)** EM reconstructions of *Saccharomyces cerevisiae* Pex1/6 obtained in the presence of ATPγS or ADP. Top view (upper row), side view (middle row) and cut open side view (lower row) surface representations of EM maps (white) fitted with Pex1/6 homology models are shown. The color code is as follows: Pex1 NBD1, red; Pex1 NBD2, blue; Pex6 NBD1, orange; Pex6 NBD2, light blue. The table lists the electron microscopy database (EMD) accession codes, the nucleotide present during data collection, the resolution obtained, the symmetry applied during refinement, and the reference for each EM reconstruction. **(D)** Overlay of EMD-2583 (mesh) with EMD-6359 (solid). The color code of EMD-6359 is as described in **(B)**. The N-terminal domains are colored in white.

Pex1 as well as Pex6 were first both described a decade later than p97 in 1991 and 1993, respectively (Erdmann et al., 1991; Spong and Subramani, 1993). The Pex1 and Pex6 ATPase domains were instantly identified to be homologous to previously described domains in p97 and N-ethyl-maleimide sensitive factor (NSF) leading to the affiliation of Pex1 and Pex6 with the growing group of ATPases associated with diverse biological activities (Erdmann et al., 1991; Spong and Subramani, 1993). It is generally accepted, that the import of peroxisomal proteins, which in contrast to proteins of other organelles are exclusively nuclear encoded, is an ATP-driven process. Thus, being so far the only peroxins with a characterized ATPase activity elevates Pex1 and Pex6 importance in the overall homeostasis of peroxisomes. Their relevance is further highlighted by the fact that 60% and 16% of all cases of PBDs are caused by mutations in Pex1 and Pex6, respectively (Waterham and Ebberink, 2012). Initially, Pex1 and Pex6 were believed to perform complementary functions as two independent type II AAA+. This hypothesis was consolidated by findings, reporting partial rescue of certain Pex1 and Pex6 phenotypes when Pex6 and Pex1 was overexpressed, respectively. However, it was established that Pex1 and Pex6 form a heteromeric complex in an ATP - and Mg^2+^- dependent manner (Faber et al., 1998; Geisbrecht et al., 1998; Tamura et al., 1998). Although a hexameric structure has been proposed ever since the discovery of the direct interaction, it was not until 2012 when Saffian and co-workers presented evidence for a 700 kDa hexamer with a 1:1 stoichiometry (Saffian et al., 2012). The first structures, confirming the formation of a hexamer with alternating Pex1/Pex6 dimers, were published in 2015 (Figure 1; Blok et al., 2015; Ciniawsky et al., 2015; Gardner et al., 2015).

## ATPase activity of the Pex1/6 complex

In contrast to p97, closer examination of the amino acid sequence reveals only 28–30% identity and substantial sequence variations between the two NBD in Pex1 and Pex6. Both proteins contain a weakly conserved Walker A and Walker B motif in the first NBD (Figure 1A). The Pex1 Walker B motif shows an exchange of the conserved glutamate residue involved in ATP hydrolysis to asparagine and aspartate in yeast and humans, respectively (Beyer, 1997; Kiel et al., 1999). The acidic residues of the Pex6 NBD1 Walker B motif and the two arginine fingers of Pex1 NBD1 are absent in most eukaryotic model organisms (Ciniawsky et al., 2015). The arginine fingers of Pex6 NBD1 are only partially conserved in the yeast but not in the human protein. In summary, NBD1 of Pex1 and Pex6 are expected to bind nucleotides, but they most probably have no ATPase activity. NBD2 of both, Pex1 and Pex6, on the other hand, shows all the characteristic conserved features including Walker A motif, Walker B motif as well the SRH, suggesting that the NBD2 of Pex1/Pex6 is responsible for the ATPase activity of the entire complex. Interestingly, neither of the NBDs of yeast Pex1 or Pex6 can oligomerize on its own (Birschmann et al., 2005). Multiple analyses have underlined the importance of the NBD2 for the overall ATPase activity of the yeast complex (Blok et al., 2015; Ciniawsky et al., 2015; Gardner et al., 2015). However, the ATPase activity is not equally split between Pex1 and Pex6 NBD2. When a Walker B mutant is introduced into Pex1 NBD2 the overall ATPase activity is reduced to 50–80% of the WT-activity in yeast. Despite the mutation, cells are still able to grow on oleate as a sole carbon source, indicating functional peroxisomes (Ciniawsky et al., 2015). Thus, ATP hydrolysis in Pex1 NBD2 is not essential for Pex1/6 function. When the same mutation is introduced in NBD2 of Pex6 the ATPase activity of the complex is completely abrogated (Ciniawsky et al., 2015; Gardner et al., 2015), emphasizing the cooperativity between the peroxins in the hexamer. In contrast to p97, very little *in vitro* data is available describing Pex1 and Pex6 ATPase activity. The only data published so far, show an apparent Km for ATP binding to yeast Pex1/Pex6 ranging from 0.17 to 0.69 mM (Saffian et al., 2012; Gardner et al., 2015), magnitudes different from the binding affinity of p97 toward ATP.

## Interaction between the Pex1/6 complex and Pex5 or Pex26

Two potential interacting partners have been identified for Pex1/6: Pex5 and Pex26 (Pex15 in yeast; Birschmann et al., 2003; Platta et al., 2005, 2008; Tamura et al., 2006). Pex5 recognizes proteins that carry a peroxisomal targeting sequence and delivers them to the peroxisomal membrane. Not only can it be found in the cytosol but it is also incorporated into the membrane of peroxisomes, where it is proposed to form a temporary protein conducting channel (Erdmann and Schliebs, 2005). The Pex1/6 complex is responsible for ATP dependent recovery of the ubiquitinated Pex5 from the peroxisomal membrane (Platta et al., 2005), although a direct binding between Pex1/6 and Pex5 could not be reconstituted so far. It is plausible that the Pex1/Pex6 complex either recognizes ubiquitinated Pex5 or, as well as p97, needs adaptor proteins to interact with Pex5. Similar to p97, ubiquitin or ubiquitin like domains are suspected to bind to one of the four N-terminal domains of the complex. Intriguingly, Pex1 and Pex6 harbor two N-terminal domains, N1 or N2, each similar to the N-terminus of p97 and NSF (Figure 1B) (Shiozawa et al., 2004; Blok et al., 2015). Pex26 is permanently anchored to the peroxisomal membrane and has been shown to bind to the N-terminus of Pex6 when both Pex6 NBDs are ATP bound (Matsumoto et al., 2003). Dissociation from the membrane anchor is mediated by ATP hydrolysis in Pex6 NBD2 in yeast and human cells. Intriguingly, it has been shown that the interaction with the cytosolic part of Pex15 (Yeast homolog of Pex26) greatly reduces the ATPase activity of the NBD2-domains (Gardner et al., 2015). This suggests that the ATPase activity is spatially and temporarily regulated to ensure energy efficient retraction of Pex5 from the membrane only upon substrate binding to Pex1/6. The exact mechanism remains rather elusive and requires a more detailed mechanistic and structural understanding of the complex. Current models interpreting the collective data suggest that the ATPase activity of the Pex1/Pex6 complex results either in a partial or complete unfolding of the membrane anchored Pex5, thereby releasing the protein to be refolded or degraded by the 26S proteasome (Platta et al., 2005, 2008). This process is similar to p97 assisted ERAD, where p97 is required for extraction of luminal as well as membrane proteins from the ER upon their labeling with ubiquitin. The extracted protein is subsequently degraded by the 26S proteasome. Similarly to the Pex6-Pex26 interaction, p97 requires membrane-embedded ERAD components to be localized to the ER. While p97 can extract a variety of ubiquitinated proteins from the ER, Pex1/Pex6 has shown to be involved in the extraction of membrane bound Pex5 only, although Pex5 and p97 substrates share ubiquitin as a common recognition motif.

## EM structures of yeast Pex1/6

Until 2015, the only available structural information on the Pex1/6 complex, was a crystal structure of the N1 fragment (amino acids 13-179) of mouse Pex1 (Shiozawa et al., 2004). Despite low sequence identity of 22% between the mouse p97 and Pex1 N-terminal fragments, both structures share the same double Ψ-β barrel fold (Figure 1B). In 2015, three groups reported first electron microscopy (EM) structures on the yeast Pex1/6 complex (Figure 1C; Blok et al., 2015; Ciniawsky et al., 2015; Gardner et al., 2015). The overall layout of the complex is identical in all three studies. Pex1 and Pex6 form a heterohexamer composed of a trimer of Pex1/6 dimers. Due to an irregular arrangement of the Pex1/6 N-terminal domains, the complex has a triangular appearance. Yet, the NBD1 and NBD2 domains form hexameric rings, which are stacked on top of each other and both of which contain a central pore of varying diameter. Two of the three structure analyses, used negative stain EM and obtained 3D reconstructions of 17–23 Å resolution (Ciniawsky et al., 2015; Gardner et al., 2015). The third analysis used cryo EM to solve Pex1/6 structures in the presence of ATPγS and ADP yielding 7.2 and 8.8 Å resolution, respectively (Figure 1C). None of the structures is of sufficient resolution to allow for unambiguous assignment of the nucleotide being bound to the binding pockets, and therefore heterogeneous binding cannot be ruled out. The studies that used negative stain EM investigated the Pex1/6 structure in the presence of different nucleotides (ATPγS, ADP or ATP, Gardner et al., 2015 and ATPγS, ATP, ADP, ADP-AlFx, Ciniawsky et al., 2015) or mutations (Pex1/6DWB, Pex1/6WB, Pex1WB/6, Ciniawsky et al., 2015). Figure 1C shows the EM structures of all groups in the presence of ATPγS or ADP. In one case, complex formation in the presence of ADP was poor leading to a small EM dataset and a poorly defined map (Ciniawsky et al., 2015). We therefore include the EM map obtained in the presence of the post hydrolysis transition state analog ADP-AlFx instead of ADP to the comparison (Figure 1C). The reconstructions in the presence of ATPγS have a very similar overall architecture. Despite the difference in resolution between maps obtained by cryo EM and negative stain, overlay of the structures demonstrates that the domain orientation is almost identical (Figure 1D). When homology models of the NBD1 and NBD2 domains are fitted as rigid bodies into the low resolution maps, the secondary structure elements overlay well with visible alpha helices and beta sheets of the higher resolution maps.

All three studies have in common that very little to no density for Pex1-N1 can be detected in the EM maps, suggesting that this domain is flexibly attached to Pex1-N2. Only one study shows significant Pex1-N movements between pre-hydrolysis and post-hydrolysis states at low resolution (Ciniawsky et al., 2015), which hint at a directed movement of the Pex1 N-terminus. The cryo EM reconstructions show little structural differences between the two examined nucleotide states, making it impossible to deduce the functional dynamics of nucleotide hydrolysis. The negative stain reconstructions on the other hand show significant structural changes between the different examined nucleotide states. Between the ADP and ATPγS bound structures, Gardner and colleagues report (1) a rotation of the NBD2 ring relative to the NBD1 ring, (2) a rearrangement of the nucleotide binding domains in NBD1 and NBD2, and (3) a narrowing of the NBD2 pore. Ciniawsky and colleagues also show distinct NBD movements in dependence of the nucleotide or mutation present. In particular, a downward rotation of NBD2 is observed when ADP-AlFx or ADP are present or when either the NBD2 of Pex1 or Pex6 carries a Walker B mutation presumably inducing a post hydrolysis state in some NBD2 binding sites. Furthermore, the structures suggest a nucleotide dependent contact between NDB2 of Pex6 and NBD1 of Pex1. Finally, the study shows that Pex1 NBD2 is locked in a post-hydrolysis state when Pex6 NBD2 is permanently bound to ATP, indicating that Pex1 NBD2 can undergo one round of ATP hydrolysis under these circumstances. Since all studies demonstrate that the Pex1/6 complex has no ATP hydrolysis activity when NBD2 of Pex6 carries a Walker B mutation (Table 1), these structural results suggest that Pex1 NBD2 is unable to release ADP or bind ATP in this Pex6 NBD2 Walker B mutant.

In summary, recently published EM structures of the yeast Pex1/6 complex agree in the overall architecture of the complex, but a common mode of action cannot be deduced from these works. While some studies observe domain movements in the whole complex (Ciniawsky et al., 2015; Gardner et al., 2015), others detect no movements whatsoever upon ATP hydrolysis (Blok et al., 2015). In particular domain movements in the N-terminal domains and NBD2 are reminiscent of nucleotide dependent movements observed for p97.

## Cryo EM structures of eukaryotic and archaeal p97

**Figure 2:**
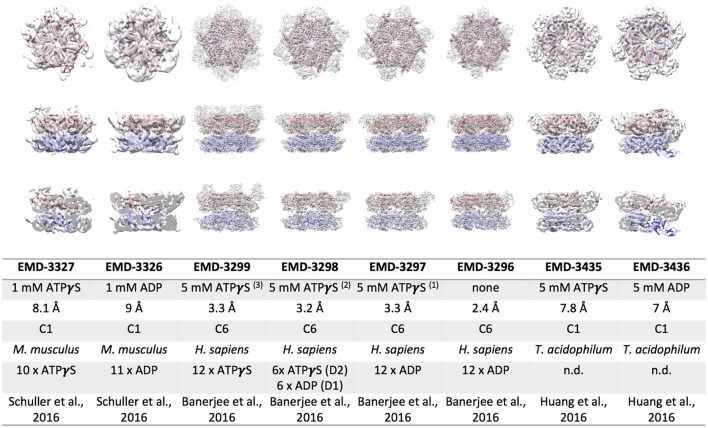
**Cryo EM reconstructions of p97/VAT obtained in the presence of ATPγS or ADP**. Top view (upper row), side view (middle row) and cut open side view (lower row) surface representations of EM maps (white) fitted with the respective p97 model are shown. The color code is as follows: p97 NBD1, red; p97 NBD2, blue. The table lists the electron microscopy database (EMD) accession codes, the nucleotide present during data collection, the resolution obtained, the symmetry applied during refinement, the source organism, the number nucleotides bound to the complex, and the reference for each EM reconstruction.

P97 is by far the best studied and best characterized ATPase among the exciting AAA+ protein family. Hence the visualization of the p97 complex at a high resolution in the most natural conditions possible has been a pressing issue over the past years. The organization of the protein complex has been intensively studied using many different structural techniques including X-ray crystallography, small angle X-ray diffraction (SAXS) and Cryo-EM. The protein data bank itself contains numerous structures of p97. Despite this, the nucleotide dependent dynamics of the complex remain elusive, because full length X-ray crystallographic structures reveal very little structural changes between AAA+ assemblies in different nucleotide bound states. In brief, three different models for p97 segregation activity are currently considered (Buchberger, 2013) (i) threading of substrate molecules through a central channel of the complex formed by aromatic residues in the pore loops (ii) substrate processing by aromatic residues in the interior of the NBD2 ring without substrate passage through the NBD1 pore, and (iii) large scale movements of the N-terminal, substrate binding domains. Cryo EM structures of p97 deposited prior to 2016 are all resolved to ~15 Å resolution and although they indicated nucleotide dependent structural re-arrangements they were of too low resolution to elucidate p97 molecular interactions. The recent advances in electron detection and image processing in cryo EM led to new efforts in structure elucidation of p97. In 2016, three groups independently published considerably improved cryo EM structures of p97/VAT (Banerjee et al., 2016; Huang et al., 2016; Schuller et al., 2016). Figure 2 shows cryo EM reconstructions of mouse p97, human p97 and an archaeal p97, called VAT, obtained in the presence of ADP or ATPγS. The resolution of the mouse and archaeal p97 structures was determined to 6–9 Å, whereas near-atomic resolution structures of 2.4–3.3 Å resolution were obtained for human p97. A distinct density for the nucleotide in the binding pocket can only be seen in the cryo EM maps of human p97. It is important however, to mention that the structures have been obtained applying six-fold symmetry and thus would not provide information about asymmetric nucleotide occupancy in the ring, which have been observed in other studies (Briggs et al., 2008; Schuller et al., 2016). The eukaryotic p97 structures both show a pronounced movement of the N-terminal domains and a rotation of the NDB2 domains upon ATPγS binding (Barthelme and Sauer, 2016; Schuller et al., 2016). Mouse p97 binds 10 ATPγS in the complex and the asymmetric reconstruction indicates that not all N-terminal domains resolved due to flexibility. N-terminal domains that are visible are rotated by ~90° and shifted by ~12.5 Å in comparison to the ADP bound state. The dataset of ATPγS bound, human p97 was subjected to 3D classification and gave three distinct classes (Banerjee et al., 2016). The resulting structures suggest that the conformational change associated with ATPγS binding can be broken down into two steps. First, the NBD2 domains are binding to ATPγS, leading to a pivot-like movement of the NBD2 domains, narrowing the NBD2 pore dimension. In a second binding event the NBD1 domains are also occupied by ATPγS, lifting the N-termini from a position coplanar to the NBD1 domains to a position significantly above the NBD1 ring, as seen for mouse p97. Furthermore, binding of ATPγS leads to a stabilization of the C-terminal peptide from residue 763–768 in human p97. Intriguingly, this observation matches recent X-ray crystallographic data of ATPγS bound human p97, showing that R766 directly contacts the gamma phosphate of the neighboring subunit in the ring (Hanzelmann and Schindelin, 2016). Mutational analysis of R766 indicates that this residue is involved in nucleotide binding and regulation of the complex's catalytic function. Altogether, the structural differences observed between eukaryotic ADP and ATPγS bound p97 EM structures agree with model (ii) and (iii), but substrate threading most likely is impossible due to a very narrow pore in the NBD1 ring. Large scale movements of the p97 N-terminal domains were previously reported in a crystallographic study of the N-NBD1 fragment carrying disease associated point mutations (Tang et al., 2010). The cryo EM structures of full length mouse and human p97 confirm that these conformational changes occur in solution upon ATPγS binding (Banerjee et al., 2016; Schuller et al., 2016).

The archaeal p97/VAT reconstructions of Huang and colleagues are the best resolved structures of this complex so far, as no atomic resolution structures of VAT are available to date. Despite having almost 50% identity at sequence level, human p97 and VAT differ in their biochemical and structural properties. VAT has been shown to associate directly with the archaeal proteasome (Barthelme et al., 2014) and to utilize pore loop residues in both NBDs for substrate remodeling (Gerega et al., 2005), suggesting substrate threading through the central pore. Structurally, the ATPγS bound EM map of VAT resembles the ADP bound structures of p97 with regard to positioning of the N-terminal domains as well as the rotation of the NBD2 domain (Figure 2; Huang et al., 2016). However, the ADP bound map of VAT considerably differs from all other p97 structures. It shows a split washer like, spiral arrangement of the subunits in the double ring that connects NBD1 of one protomer along the seam with NBD2 of the other protomer forming the seam. Similar arrangements have been observed for other remodeling AAA+ complexes, such as ClpA, Hsp104, and rubisco activase (Guo et al., 2002; Stotz et al., 2011; Yokom et al., 2016). In contrast to the eukaryotic structures, the VAT cryo EM structures agree with the mechanistic model (i), although the pore residues are arranged in a helical opening. In this case, ATP hydrolysis re-locates the NBD1 domains from a co-planar arrangement in the presence of ATPγS to the spiral arrangement in the presence of ADP, exerting differential pulling forces on various parts of the substrate (Huang et al., 2016). A nucleotide dependent movement of the N-terminal domains is not observed for VAT. A recent follow up study describes the substrate engaged ΔN VAT hexamer in different nucleotide bound states, confirming the translocation of the substrate through the central pore of ΔN VAT (Ripstein et al., 2017). Mechanistically, unfolding is proposed to be mediated by processive hand-over-hand substrate binding within the ring.

## Structural differences between p97 and Pex1/6 EM reconstructions

**Figure 3:**
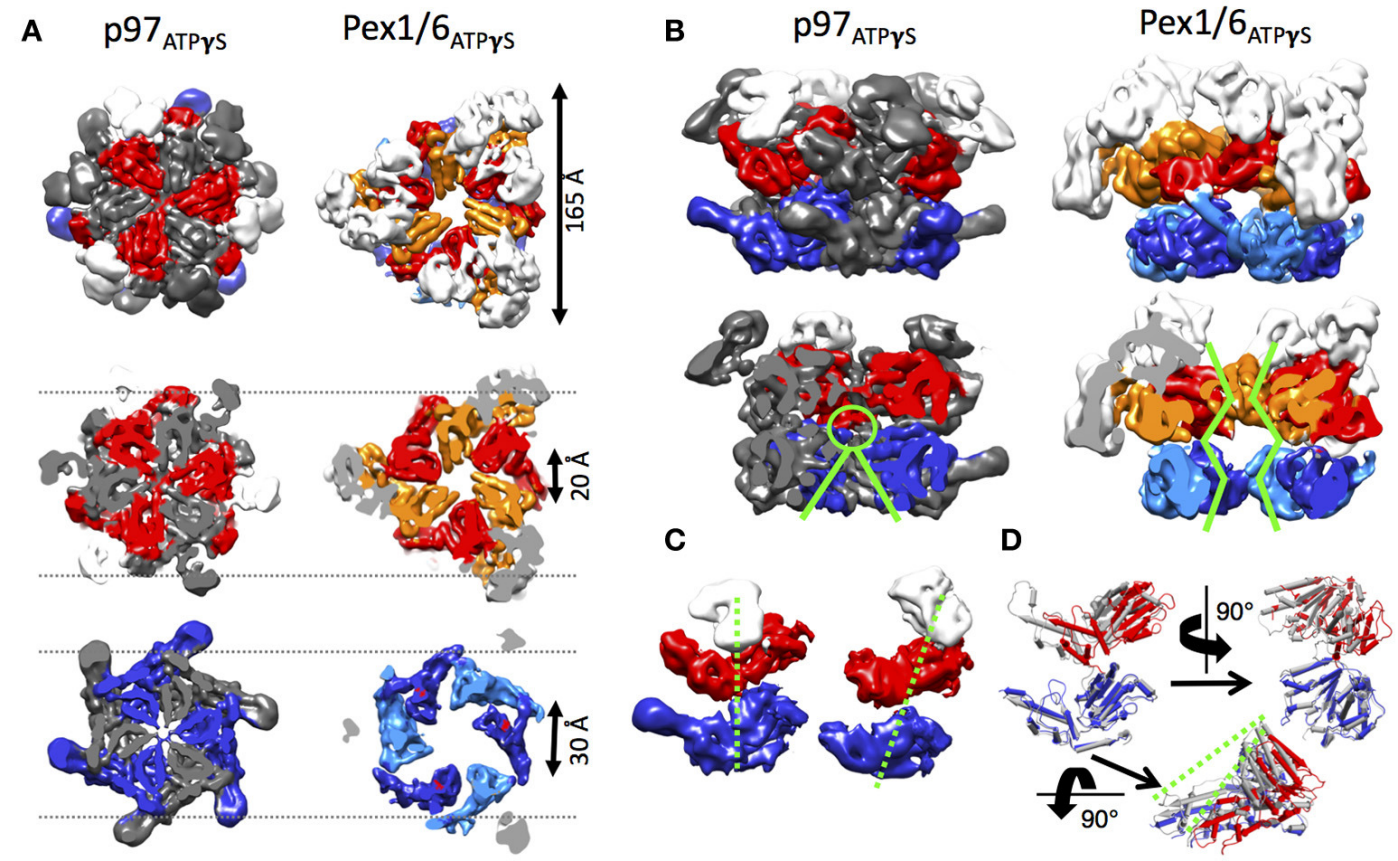
**Structural differences between p97 and Pex1/6. (A)** Side-by-side comparison of cryo EM structures of mouse p97 (EMD-3325) and yeast Pex1/6 (EMD-6359) in the presence of ATPγS. EMD-3325 is derived from the same dataset as EMD-3327, but C6 symmetry was applied during refinement. Surface representations as top view (upper row), slice of NBD1 (middle row) and slice of NBD2 (lower row) are shown. The color code is as follows: Pex1 NBD1 and p97 NBD1, red; Pex1 NBD2 and p97 NBD2, blue; Pex6 NBD1, orange; Pex6 NBD2, light blue; N-termini, white. Every second protomer of p97 is colored in gray to distinguish protomers in the rings. **(B)** Side view (top row) and cut-open side view (bottom row) surface representation of p97 and Pex1/6 as described in **(A)**. Green lines indicate pore sizes in the NBDs at different positions along the central axis. **(C)** Position of the NBDs of p97 (left) and Pex1 (right), when the complexes are overlaid on the NBD rings. Only one protomer of p97 and Pex6 is shown and color coded according to **(A)**. Domain offsets between the NBDs is indicated by green dotted lines. **(D)** NBD1/2 protomers of p97 (gray) and Pex1 (red/blue) are superimposed on their NBD2 domains. Green dotted lines indicate the rotation and shift in Pex1 NBD1 (red) in comparison with p97 NBD1 (gray).

**Figure 4:**
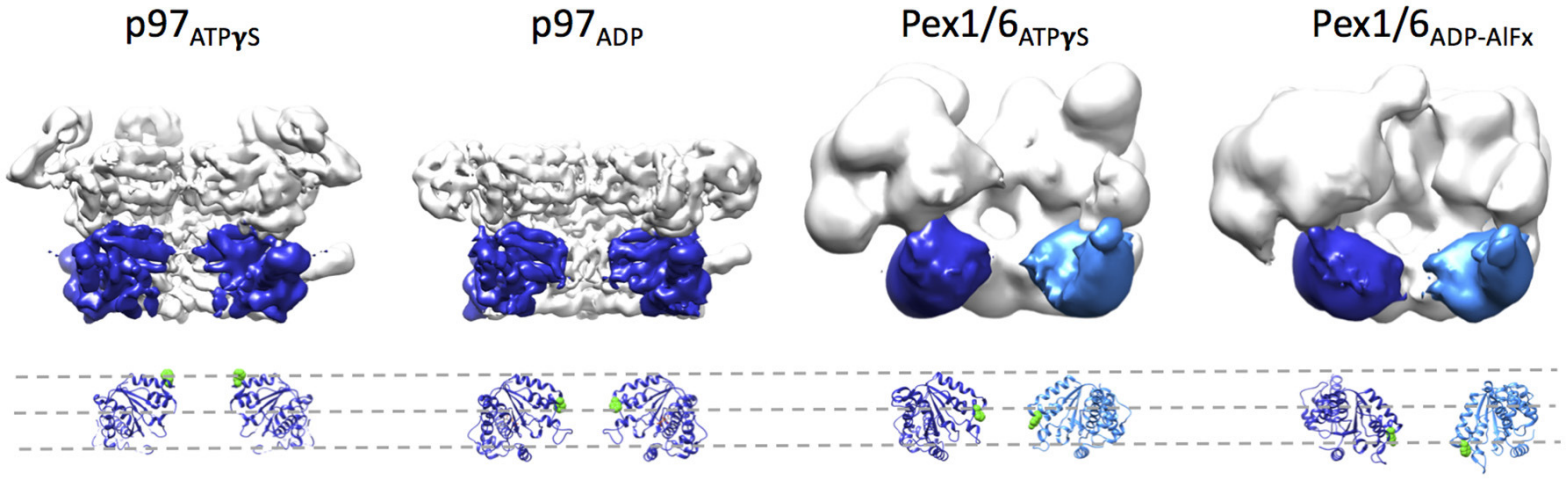
**Rotation of the NBD2 domains upon ATPγS binding**. Surface representations of p97 in the presence of ATPγS (EMD-3325) or ADP (EMD-3326) and Pex1/6 in the presence of ATPγS (EMD-2583) or ADP-AlFx (EMD-2584) are shown. Two protomers are omitted from each hexamer. Two NBD2 domains of p97 and the NBD2 domain of Pex1 are colored in blue. One NBD2 domain of Pex6 is colored in light blue. Underneath, a ribbon representation based on rigid body fits of p97 crystal structures (pdb 3CF3/ADP and 3CF2/ATPγS) or Pex1/6 homology models into the EM maps is shown. Conserved aromatic residues p97^F552^, Pex1^F771^, and Pex6^Y805^ are shown as green spheres. All structural figures were done using the UCSF Chimera package (Pettersen et al., 2004).

Side-by-side comparison of the ATPγS bound p97 and Pex1/6 EM structures of similar resolution shows that the outer diameter of the NBD rings is almost identical (Figure 3A). Nonetheless, the diameter of the inner pore in the NBD1 ring is closed in all p97 structures, while Pex1/6 NBD1 rings form around an inner pore of ~20 Å. The pore diameter in NBD2 is ~10 and ~30 Å for p97 and Pex1/6, respectively. It should be mentioned that the density in the NBD2 domains of the cryo EM structures of Pex1/6 is fragmented and that the negative stain maps indicate pore diameters of only ~10 Å for the NBD2 ring. The close arrangement of the NBD1 domains in the recent cryo EM reconstructions of eukaryotic p97 agrees with the suggestion that this complex does not thread substrates through the central axis across the length of the barrel and that substrate can only access the NBD2 pore loops by entering and exiting through the NBD2 pore end (Figure 3B). In contrast, the Pex1/6 complex provides a sizable central channel through the entire structure and would thus be consistent with a substrate threading mechanism. The Pex1/6 complex also distinguishes itself from p97 in the overall domain arrangement. While p97 NBD1 and NBD2 are almost located on top of each other in the ATPγS bound state of the complex, the NBDs of Pex1 and Pex6 show a staggered arrangement (Figures 3A,C). Accordingly, the orientation and relative location of the N2 domain of Pex1 in the ring differs from that of the p97 N-terminal domain. When NBD1/2 protomers of p97 and Pex1/6 bound to ATPγS are superimposed on their NBD2 domains the relative orientation of the AAA+ domains to each other becomes apparent (Figure 3D), revealing that Pex1 NBD1 is rotated and shifted outwards with regard to p97 NBD1. This difference in domain arrangement possibly leads to the formation of a bigger central pore in the NBD1 ring of Pex1/6. It should be noted that Gardner and colleagues observe some nucleotide dependent rotation of the Pex1/6 AAA+ rings against each other (Gardner et al., 2015), although the staggered arrangement persists in all nucleotide states. Whether or not the two AAA+ domains adopt a stacked or staggered arrangement in the hexamer might be influenced by the peptide linker that connects the AAA+ domains. Typically, the linker region is not very well conserved and the sequence can differ greatly between different type II AAA+ complexes. So far, no prevalent signal transduction pathway has been found that involves the linker between the AAA+ domains or the linker that connects the N-terminal domains to NBD1. However, the linker region between the N-terminal domain and NBD1 of p97 has been shown to be prone to disease associated mutations (Watts et al., 2004), which seem to trap p97 in a state comparable to the ATPγS bound state (Tang et al., 2010; Tang and Xia, 2013), presumably by preventing movement of the N-terminal domains. Furthermore, an atomic resolution cryo EM structure of human p97 bound to ADP as well as the allosteric inhibitor UPCDC30245 (Banerjee et al., 2016) indicates that some degree of flexibility at the interface of the AAA+ domains is needed for the rotational movement of NBD2. This nucleotide dependent, rotational movement in NBD2 has been observed for human and mouse p97 as well as for yeast Pex1/6 (Figure 4). In all cases nucleotide hydrolysis triggers a rotation of the NBD2 domain that moves the substrate binding loops from a central position to a position closer to the C-terminal opening of NBD2. Interestingly, the most common missense allele in human Pex1, *Pex1G843D*, causes an amino acid exchange at the interface between NBD1 and NBD2 in Pex1, possibly disturbing complex dynamics. In summary, comparison between the cryo EM maps of p97 and Pex1/6 reveals variations in stacking of the tandem AAA+ domains in the complexes, possibly resulting in a larger central channel formed by Pex1/6. The EM structures of eukaryotic p97 exclude substrate threading through the entire length of the AAA+ double layer, due to a very narrow NBD1 pore. The yeast Pex1/6 structures on the other hand support such a mechanistic model, with some of them showing a nucleotide dependent downward rotation of the NBD2 domains (Ciniawsky et al., 2015). The structural studies on Pex1/6 have also demonstrated that the Pex1 N-terminal domains and in particular the Pex1 N1 domain are flexible. Thus, a mechanistic model, whereby Pex1/6 segregates or dislocates substrate protein by movement of the N-terminal domains is also plausible.

## Outlook

Despite a variety of new structural information on type II AAA+ complexes, such as p97 and Pex1/6, we still have a rather static view on these multiprotein assemblies. In order to elucidate their nucleotide dependent dynamics, structural analysis of the complexes in different nucleotide bound states, biochemical findings and cell biological data need to be interconnected. Structure determination of these heterogeneous complexes to below ~4 Å resolution to allow for identification of the nucleotide bound to the NBDs is still a challenging task. The derivation of a common mode of action is further complicated by divergent structural and biochemical features of homologous proteins. Thus, a comprehensive characterization of each AAA+ complex is needed, before we can distinguish between specialization and similarities in force generation of different AAA+ proteins. Some of the many questions that remain to be answered for p97 and Pex1/6 complexes are (1) how variations in the structure of the AAA+ domain translate into functional differences, (2) what is the molecular basis of substrate remodeling, (3) how does substrate binding to the complex influence AAA+ activity, and (4) how is the interplay between orientation of the N-terminal domains and AAA+ binding status regulated.

## Author contributions

All authors listed, have made substantial, direct and intellectual contribution to the work, and approved it for publication.

### Conflict of interest statement

The authors declare that the research was conducted in the absence of any commercial or financial relationships that could be construed as a potential conflict of interest.
